# Struma ovarii mimicking ovarian malignancy: a case report

**DOI:** 10.1093/jscr/rjaf439

**Published:** 2025-06-23

**Authors:** Giulia Bruni, Marta Tripepi, Ana Gomes da Costa, Adalgisa Guerra, Ana Catarino, João Casanova

**Affiliations:** Department of Medical Sciences, University of Ferrara, Ferrara 4421, Italy; Unit of Gynecology and Obstetrics, Department of Women and Children, University of Padua, 35122 Padua, Italy; Gynecologic Oncology Unit, Obstetrics and Gynecology Service, Department of Surgery, Hospital da Luz de Lisboa, Lisboa, Portugal; Radiology Department, Hospital da Luz Lisboa, Avenida Lusíada, N° 100, 1500-650 Lisbon, Portugal; Department of Pathology, Hospital da Luz Lisboa, Avenida Lusíada, N° 100, 1500-650 Lisbon, Portugal; Gynecologic Oncology Unit, Obstetrics and Gynecology Service, Department of Surgery, Hospital da Luz de Lisboa, Lisboa, Portugal

**Keywords:** struma ovarii, ovarian tumor, ascites, CA125, teratoma, ovarian cancer

## Abstract

Struma ovarii is a rare form of monodermal ovarian teratoma composed predominantly of mature thyroid tissue. Although benign in most cases, it can present with ascites and elevated serum CA125 levels, mimicking ovarian carcinoma. We report the case of a 38-year-old woman with a complex ovarian mass, ascites, and an elevated CA125 level—an initial presentation highly suggestive of malignancy that was ultimately diagnosed as benign struma ovarii following surgical intervention. This case underscores how struma ovarii, though uncommon, can masquerade as ovarian cancer and may even be discovered incidentally in an asymptomatic, premenopausal patient.

## Introduction

Struma ovarii is a rare form of monodermal teratoma, accounting for <1% of all ovarian tumors [1], and is defined by thyroid tissue comprising >50% of the tumor mass [2]. While most cases are benign and asymptomatic, struma ovarii may present with clinical and radiological features that closely mimic ovarian carcinoma—especially in the presence of ascites, elevated tumor markers, and complex adnexal masses. Here, we present the case of an asymptomatic 38-year-old woman who was ultimately diagnosed with struma ovarii following a clinical and radiologic workup that strongly suggested an ovarian malignancy.

## Case report

A 38-year-old woman presented for a routine gynecological evaluation. She was completely asymptomatic and denied any weight loss, abdominal pain or distension, gastrointestinal disturbances, early satiety, or menstrual irregularities. Her past medical history was unremarkable except for having undergone a laparotomy a few years earlier for a left ovarian cyst, which was histologically confirmed as a mature teratoma. Her family history was notable only for a father with prostate cancer.

On examination, the patient was found to have a distended abdomen, raising suspicion of ascites. Laboratory results revealed a markedly elevated serum CA125 level (1716 U/ml). Imaging studies were promptly undertaken, including a magnetic resonance imaging (MRI) of the pelvis and a contrast-enhanced computed tomography (CT) scan of the chest, abdomen, and pelvis.

**Figure 1:**
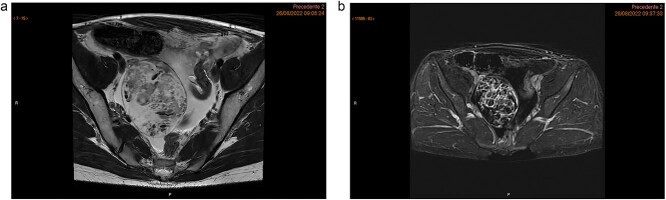
Pelvic MRI highlighting a heterogeneous right ovarian lesion with high signal on axial T2 WI (a), and irregular enhanced septum on DCE T1 WI (arrow). Ascites and a pelvic nodule with fat and contrast enhancement (arrow head) (b).

**Figure 2:**
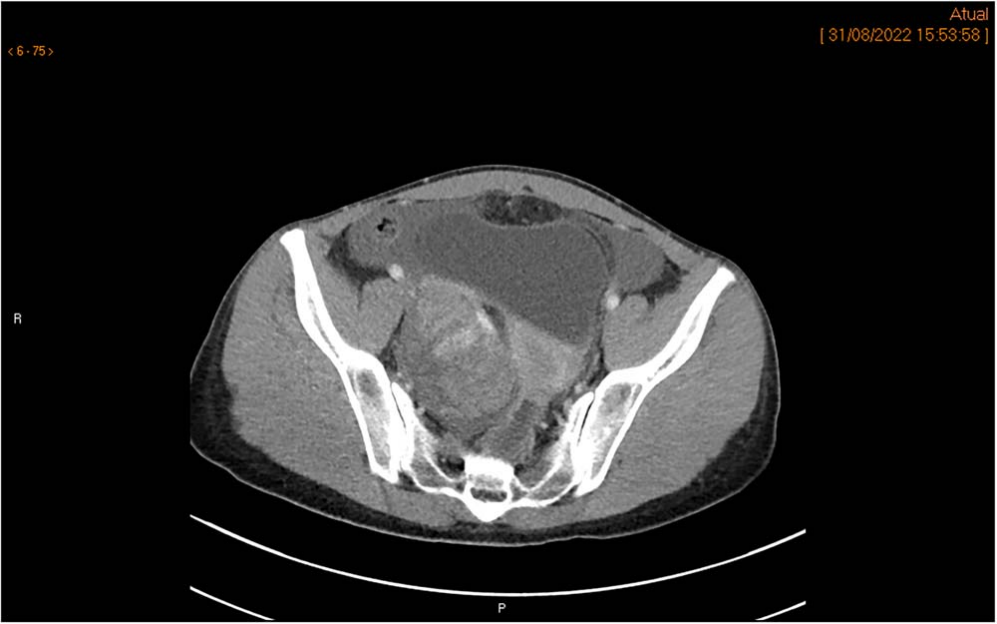
CT scan demonstrates a right ovarian mixed lesion measuring 80 × 67 × 57 mm, with solid, contrast-enhancing components, ascites, and prevesical fat stranding nodule, with no other categoric signs of carcinomatosis.

The MRI showed a heterogeneous right adnexal mass with mixed solid and cystic components. Moderate ascites was present in the subphrenic and paracolic gutters as well as the pelvic recesses. No suspicious lymphadenopathy or peritoneal thickening was observed on MRI (Fig. 1a and b). The subsequent CT scan confirmed a complex right ovarian lesion measuring approximately 80 × 67 × 57 mm, with solid contrast-enhancing components, highly suspicious for malignancy. Ascites and an indeterminate omental nodule were also noted. Additionally, subtle fat stranding in the omentum (in the lower portion of the greater omentum adjacent to the bladder), along with the moderate ascites, suggested early peritoneal carcinomatosis (Fig. 2).

**Figure 3:**
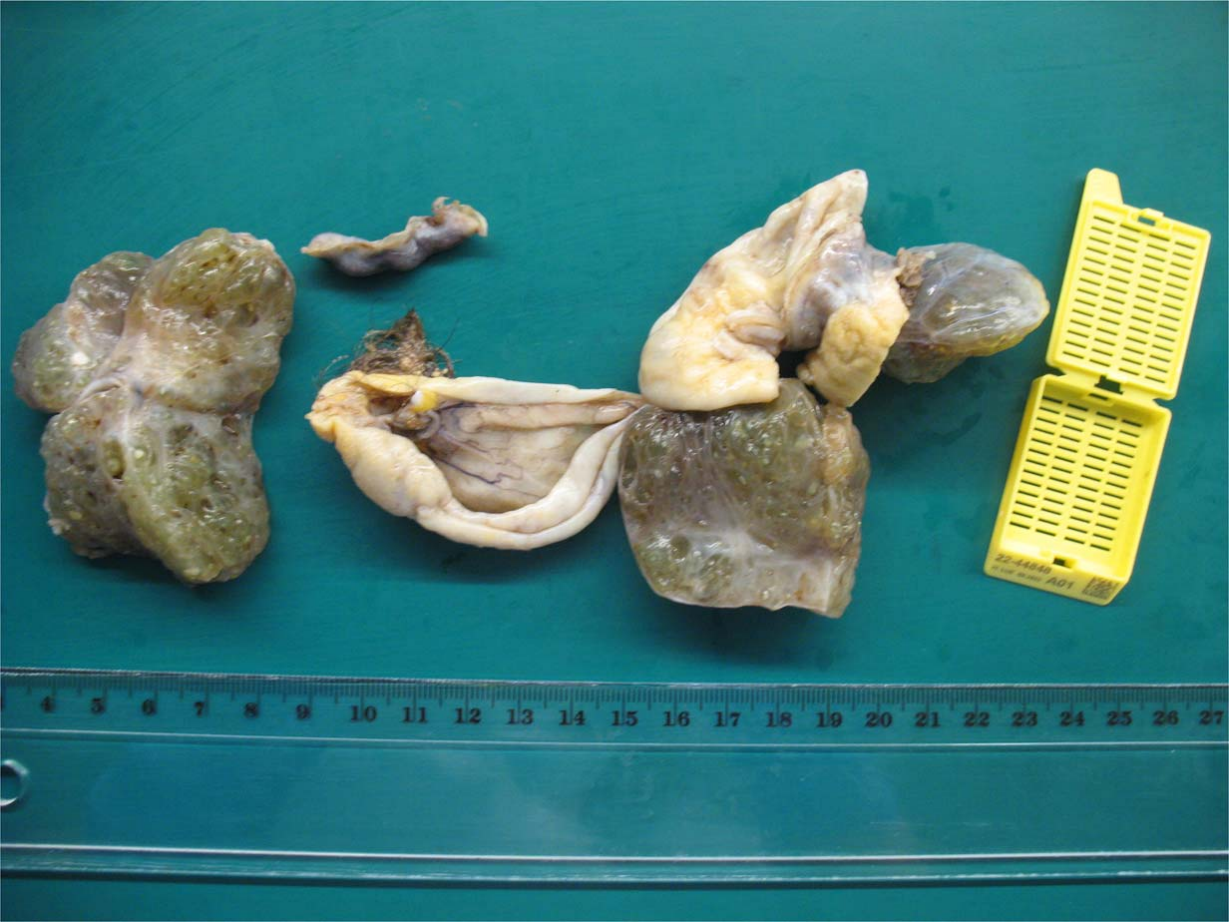
The thyroid tissue is the dominant component, along with a cyst with sebaceous material and hair.

Given the clinical presentation and imaging findings concerning for ovarian carcinoma with peritoneal involvement, the patient was scheduled for exploratory surgery. Intraoperative evaluation included a frozen section of the right tube and ovary, which revealed a benign lesion consistent with struma ovarii. The surgical procedure comprised a right salpingo-oophorectomy, excision of a left ovarian cyst, and an appendectomy (performed because the appendix appeared mildly dilated). The post-operative course was uneventful, and the patient was discharged on post-operative Day 6.

**Figure 4:**
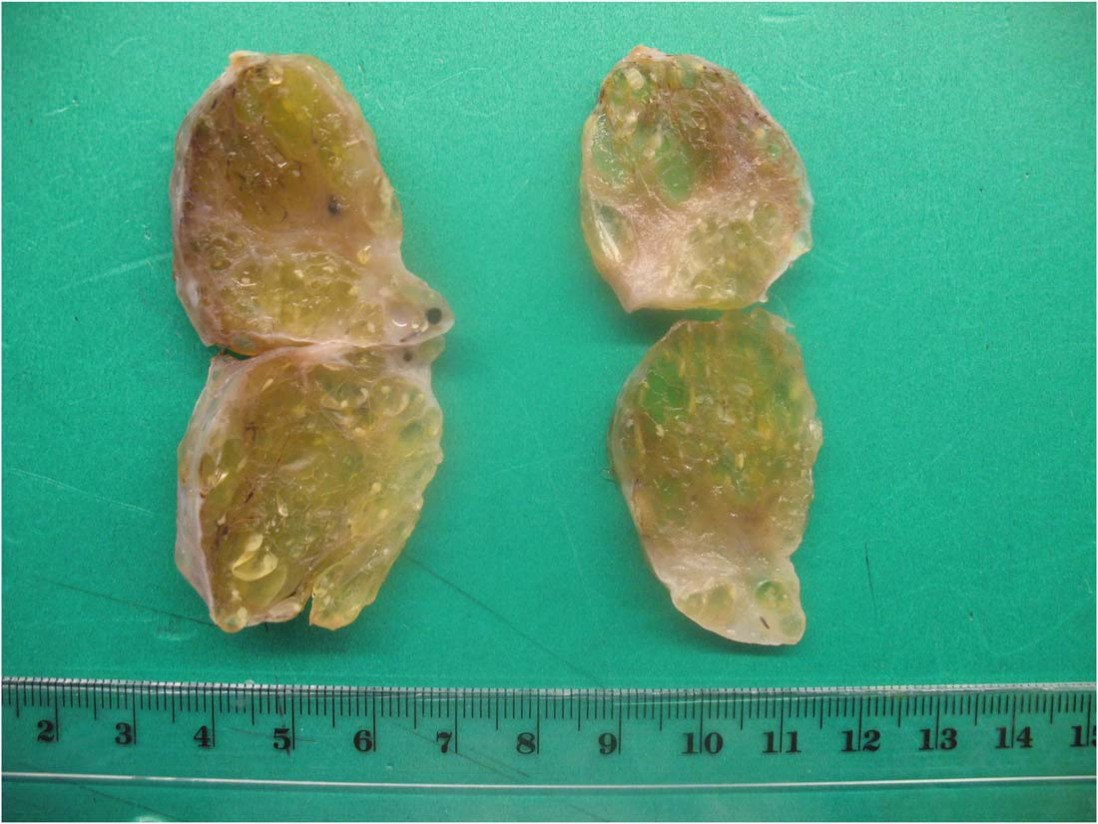
The sectioned surface is solid and cystic with a gelatinous, pale-yellow appearance (goiter-like).

**Figure 5:**
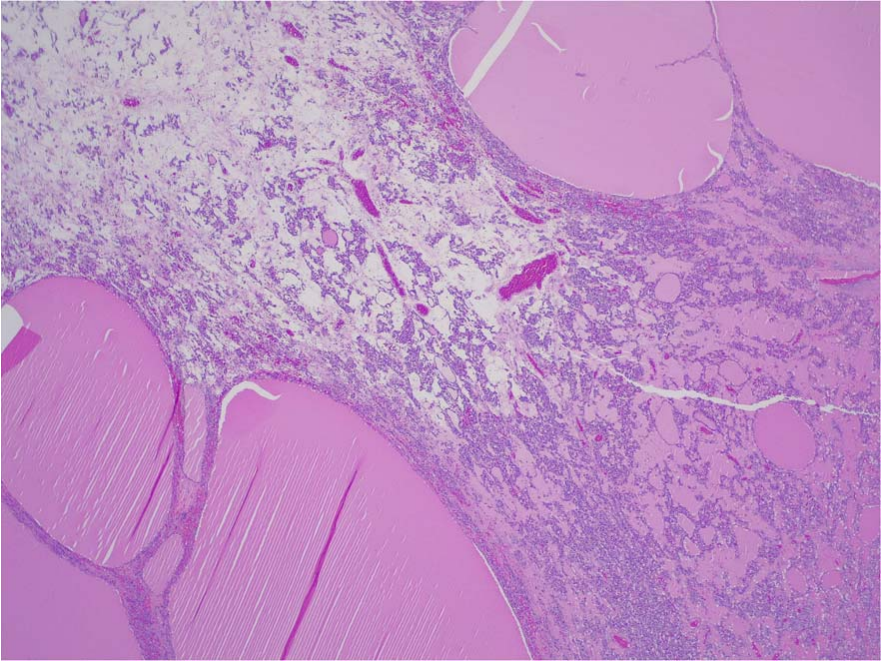
Struma ovarii—thyroid tissue is the dominant component with large and small colloid-containing follicles: H&E ×25.

**Figure 6:**
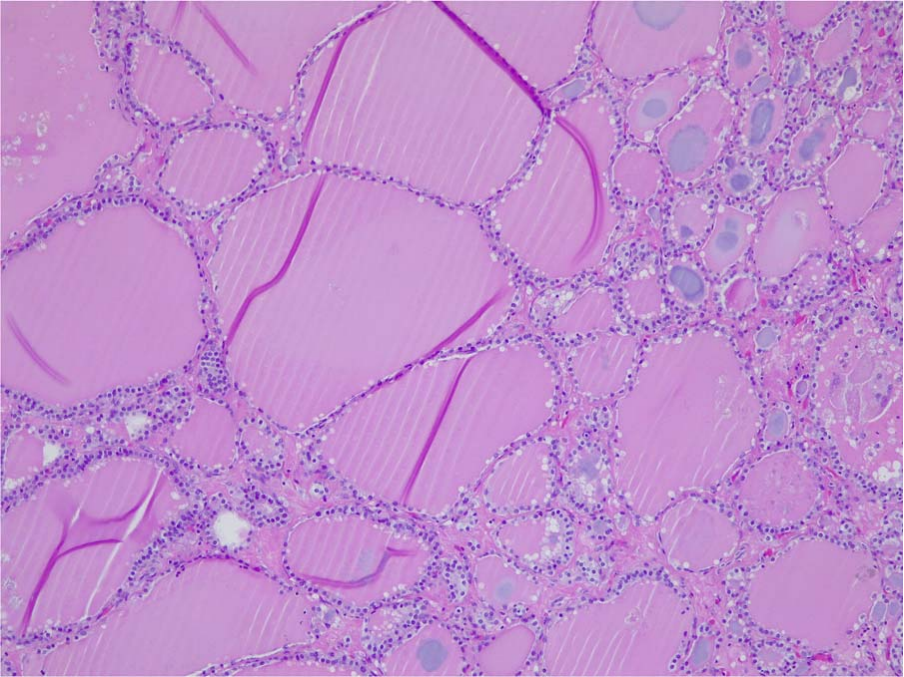
Struma ovarii—small and large thyroid follicles; H&E ×100.

Final histopathological examination confirmed the diagnosis of struma ovarii. On gross examination, the tumor was composed predominantly of thyroid tissue and contained a cyst filled with sebaceous material and hair (Fig. 3). The cut surface of the ovarian mass was partly solid and partly cystic, with a gelatinous, pale-yellow, goiter-like appearance (Fig. 4). Microscopically, the lesion demonstrated thyroid follicles of varying sizes filled with colloid (Figs 5 and 6), with no evidence of malignant transformation. The patient recovered well and has remained asymptomatic under regular follow-up. At 3 years post-surgery, she shows no evidence of disease.

## Discussion

Struma ovarii can closely resemble an ovarian malignancy both clinically and radiologically, particularly when it presents with the triad of ascites, a complex pelvic mass, and elevated CA125 levels [3]. Several cases in the literature have documented struma ovarii mimicking ovarian cancer under such circumstances [6–10]. Due to its rarity, establishing a preoperative diagnosis of struma ovarii is challenging. Imaging findings are often non-specific: ultrasound and CT typically show a complex unilateral adnexal lesion, which is not pathognomonic for struma ovarii [4]. MRI, however, may provide additional clues. Struma ovarii often contains multiple cystic locules with variable signal intensities, reflecting the heterogeneous accumulation of thyroglobulin and thyroid hormones within the lesion. Notably, thick gelatinous colloid in larger thyroid follicles can manifest as low-signal-intensity areas on both T1- and T2-weighted MRI sequences [5]. These MRI characteristics, while suggestive, are still not exclusive to struma ovarii and thus the definitive diagnosis is frequently made only through histopathological examination [5].

In most reported cases of struma ovarii that mimic malignancy, the patients were postmenopausal and symptomatic—commonly presenting with abdominal pain, distension, and/or weight loss [6–9]. In such scenarios, the clinical suspicion of an ovarian carcinoma prompts surgical exploration and intraoperative diagnosis. Interestingly, only one reported case involved a younger patient (31 years old) who presented with pelvic pain and elevated CA125 levels [10]. Our case is unusual in that the patient was a 38-year-old premenopausal woman who was entirely asymptomatic, and her ovarian mass was an incidental finding during a routine exam. This subclinical presentation highlights that struma ovarii can occur without the typical signs or symptoms, even in a patient of reproductive age. Such variability in clinical presentation underlines the importance of including struma ovarii in the differential diagnosis of ovarian masses, regardless of patient age or menopausal status. Recognizing this entity can prevent misdiagnosis and ensure that patients receive appropriate surgical management and counseling.

## Conclusion

Struma ovarii, although rare and usually benign, should be considered in the differential diagnosis when an ovarian mass is accompanied by ascites and elevated tumor markers. This case illustrates that even an asymptomatic, premenopausal woman can harbor struma ovarii that mimics advanced ovarian cancer. Early surgical intervention with intraoperative pathological assessment was crucial in our patient to obtain the correct diagnosis and avoid more extensive surgery. Awareness of this rare entity and its potential presentations can aid clinicians in managing similar cases and in reassuring patients when a benign outcome is confirmed.
